# Multi-Response Optimization and Predictive Modeling of Drilling Performance in PEEK-CF30 Composites Considering Drill Coating and Cutting Parameters

**DOI:** 10.3390/polym18091064

**Published:** 2026-04-28

**Authors:** Mustafa Günay, Mehmet Boy, Mehmet Erdi Korkmaz

**Affiliations:** 1 Department of Mechanical Engineering, Karabük University, Karabük 78050, Turkey; 2 TOBB Vocational High School, Karabük University, Karabük 78050, Turkey; mboy@karabuk.edu.tr; 3 Department of Mechanical Engineering, Yildiz Technical University, Istanbul 34349, Turkey; mehmeterdi.korkmaz@yildiz.edu.tr; 4 Information Technologies Research and Application Center, Istanbul Ticaret University, Istanbul 34445, Turkey

**Keywords:** PEEK-CF30, drilling, coating, energy consumption, multi-response optimization

## Abstract

Carbon fiber-reinforced thermoplastic composite drilling is a secondary manufacturing process because the quality of drilled holes affects assembly system performance, structure, and sustainability. This paper compares all drill coating types and cutting conditions for PEEK-CF30 composite drilling utilizing a hybrid experimental–statistical method. DLC-, TiN-, and TiCN-coated HSS drills, as well as cutting speed and feed rate were tested using the Taguchi L27 design. Performance indicators were measured by including thrust force, surface roughness, drilling torque, and energy consumption. Experimental results showed that increasing cutting speed and feed rate increased the thrust force while decreasing torque and energy consumption. Smearing on the hole surface, chip adhesion, and short fiber adhesion/pull were identified as indicators of poor surface quality, and these occurrences increased with increasing drill coating removal at high cutting parameters. In terms of overall performance, the TiCN-coated drill created the lowest thrust force (50.85 N), surface roughness (1.038 µm), torque (17.54 Ncm), and energy consumption (136.45 J) at high feed conditions. Taguchi-based gray relational analysis methodology revealed that the TiCN-coated drill, a cutting speed of 40 m/min, and a feed rate of 0.1 mm/rev are the optimum parameters. Second-order prediction models developed for all responses proved to have high predictive capabilities with coefficients of determination above 94%. Ultimately, drill coating quality considerably affected surface integrity and drilling energy consumption performance in drilling PEEK-CF30. A hybrid optimization and modeling framework demonstrates that the drill quality cutting parameter will allow for optimum selection to ensure efficient processing of advanced thermoplastic composites.

## 1. Introduction

Due to their higher specific strength, corrosion resistance, dimensional stability, and superior damage tolerance than many conventional structural materials, carbon fiber-reinforced thermoplastic composites have attracted increasing interest in advanced engineering applications [1]. Of these materials, polyether ether ketone (PEEK)-based carbon fiber (CF)-reinforced composites hold significant potential in high-performance sectors, such as aerospace, automotive, biomedical, and precision engineering, owing to their outstanding thermal stability, chemical resistance, and fatigue performance [2]. Together with these attractions, the increasing industrial inclination towards the use of thermoplastic matrix composites, as compared to thermoset-based systems, is closely linked to their recyclability, weldability, shorter processing cycles and higher impact resistance [3]. Such reasons make PEEK-CF composites an increasingly attractive technology for lightweight, robust structural components where high mechanical performance and processing flexibility are required [4].

In spite of these strengths, the machining of CF-reinforced composites still proves difficult [5]. Drilling is a necessary secondary manufacturing process in many practical applications, as holes are required for assembly, fastening, joining and integration into complex structural systems [6]. The heterogeneity and anisotropic nature of CF-reinforced thermoplastic composites make the properties of drilling related to a complicated package of fiber fracture/matrix deformation, thermal softening, interfacial debonding, smearing, and tool wear [7]. Whereas the process of chip formation is mostly predictable in the case of metallic materials, the drilling of thermoplastic composites suffers from simultaneous mechanical and thermal damage modes [8]. Wear at the cutting edge can increase if the carbon fibers are short, and the thermoplastic matrix can soften when exposed to high temperatures, resulting in unstable cutting conditions and degradation of surface integrity [9]. Thus, thrust force, torque and surface roughness become highly sensitive to tool conditions and cutting parameters [10].

Among the process outputs in composite drilling, thrust force is the most important because it is related to tool–workpiece interaction intensities as well as the amount of damage induced during penetration [11]. Increased rates of matrix cracking, fiber pull-out, local deformation, and dimensional errors are associated with excessive thrust force [12]. The surface roughness is also important to the overall response because it indicates the final hole quality that significantly impacts the overall operation (assembly, frictional behavior, and service) of the drilled equipment [13]. Drilling torque is also worth considering not only because it offers the potential for understanding chip formation resistance in terms of cutting stability and frictional conditions at the tool–material interface, but also because it is a potential parameter in the determination of tool–workpiece interaction [14]. Besides these well-known quality indicators, energy consumption has recently been established as a vital machining response from the perspective of sustainable manufacturing [15]. While energy analyses are extensive in the metal cutting literature, studies investigating energy consumption along with force, torque, and surface quality in drilling of high-performance thermoplastic composites are limited [16]. Hence, a comprehensive evaluation of drilling performance is required, especially with respect to advanced polymer composites whose thermal nature and surface integrity are closely integrated [17].

The drilling performance of such composites is greatly determined by the tool material and coating quality used [18]. The abrasive behavior of the carbon fibers encourages flank wear, edge rounding and coating degradation, while the thermoplastic matrix may adhere, smear or thermally soften under different cutting environments [19]. As a result, the tribological aspect of the tool–composite interface is strongly regulated by the coating type [20]. Coatings like diamond-like carbon (DLC), titanium nitride (TiN), and titanium carbonitride (TiCN) are extensively used to enhance cutting surface quality through their specific hardness, friction actions, and wear resistance properties [21]. Yet, their effectiveness cannot be extrapolated independently of cutting speed and feed rate [22]. A coating that shows good results at low thermal load may not hold its advantage in hard abrasive and thermal conditions, and another coating may show higher edge preservation and hole quality under the same cutting conditions. This implies that the type of drill coating should not be considered only a tool property, but rather a process variable that directly affects cutting parameters and response outputs [23].

The studies on machining polymer composites have indicated substantial impact of cutting speed and feed rate on thrust force, torque, cutting tool wear, and hole surface deterioration [24]. Generally, increase in feed rate can raise undeformed chip load and drilling resistance [25], but increased cutting speed could increase machinability to the extent that the thermal softening is balanced against the abrasive damage [26]. This is particularly tenuous when dealing with machining of thermoplastic composites because the matrix is temperature-sensitive and the reinforced fibers remain extremely abrasive [27]. While numerous studies have addressed drilling-induced damage or delamination for fiber-reinforced composites due to drilling [28], relatively few studies have focused specifically on PEEK-CF30 composites with equal emphasis on drill coating quality, multiple machining responses, and energy consumption in one analytical approach [29]. These processing indicators (responses) are usually considered individually; in this case, a common multi-response optimization problem arises, where improving one indicator might have a negative impact on another [30]. However, industrial drilling practice requires a concurrent decrease in cutting force, roughness, torque, and energy consumption.

Recent research has revealed processing characteristics in drilling carbon- or glass fiber-reinforced thermoplastic composites of various sizes. However, considerable gaps remain in the comprehensive evaluation of process responses. For example, Boy [29] studied the drilling of PEEK-CF30 composites and found that higher cutting speed and feed rate led to greater thrust forces, whereas lower cutting speed with moderate feed rates produced better surface roughness at the expense of increased delamination. However, this research was aimed at uncoated HSS tools and did not incorporate torque, temperature, and energy responses, limiting a holistic approach to the procedure. Through a comparative drilling experiment of thermoplastic CF/PEKK (CF/polyether ether ketone) and thermoset CF-poxy composites, Ge et al. [31] showed that CF/PEKK produced substantially more thrust force and hole surface temperature but resulted in significantly less delamination because of its excellent interlaminar toughness. However, the study was limited to measuring only one drilling configuration and did not include surface roughness, torque, or energy consumption metrics. Later studies by Ge et al. [8] further analyzed hole-making in CF/PEKK by matching traditional drilling and helical milling and concluded that delamination is driven by a dual thermal–mechanical mechanism, the temperature being predominant for low feeds, and thrust force high feeds. While helical milling improved wall destruction, the lack of torque power and energy consumption analysis restricts the application of these results in process optimization. Similarly, Liu et al. [32] recognized a thermo-mechanically coupled finite element model, corroborated by drilling of braided CF/PEEK composites, and demonstrated that a selection of an ideal spindle speed and feed rate could effectively minimize both thrust force and temperature with a high predictive value. However, they concentrated on thrust and thermal effects without dealing with surface roughness, torque or energy consumption. Domingo et al. [33] explored the drilling of PEEK-GF30 in the cryogenic compressed air and showed that the below-zero temperatures can yield high-energy saving in combination with the acceptable thrust force. However, their investigation was on glass fiber-reinforced PEEK and lacked an evaluation surface integrity, namely surface roughness and delamination. Therefore, its implications for carbon fiber-reinforced systems cannot be easily applied.

Predictive modeling and multi-objective optimization are also very useful for very complex machining systems that require both scientific interpretation and practical parameter selection [34]. Taguchi-based experimental design can provide an efficient way to reduce the number of experimental trials, while gray relational analysis can convert multiple quality indicators with different units to a single performance index [35]. This Taguchi-based gray relational analysis (TGRA) approach is especially beneficial for identifying optimal conditions under distinct response trends [36]. On the contrary, the response surface method (RSM) is considered to be one of the most powerful statistical tools for establishing arithmetic relationships between cutting factors and process results, visualizing factor interactions and predicting intermediate response values with reasonable reliability [37]. Taken together, TGRA and RSM present a systematic and robust structure not only for determining the best optimal parameters but also for investigating how the given variables affect the drilling behavior of the polymer composites as a whole [38].

Studies in the literature generally examine the effect of tool geometry or cutting parameters on carbon/glass fiber-reinforced PEEK composites only through thrust force, delamination, or roughness, without simultaneously considering drill coating type, multiple quality responses, and energy consumption. In contrast, this article directly compares HSS drills with DLC, TiN, and TiCN coatings, treating the coating not merely as a tool property but as a fundamental process variable interacting with cutting speed and feed rate, and simultaneously optimizing thrust force, torque, surface roughness, and energy consumption. In this context, the article presents the optimum selection of coating/cutting parameter combinations for the drillability of PEEK-CF30 composites using a Taguchi gray relationship analysis-based multi-response optimization approach commonly used in machining technologies, as well as predictive models for performance indicators developed using RSM modeling. In these respects, the study holistically addresses the coating–process–response relationship specifically for PEEK-CF30 and contributes to the field by integrating a sustainable processing perspective into drilling process optimization, which is supported by multi-response optimization and predictive modeling.

## 2. Equipment and Methodology

### 2.1. Materials and Apparatus

**Figure 1:**
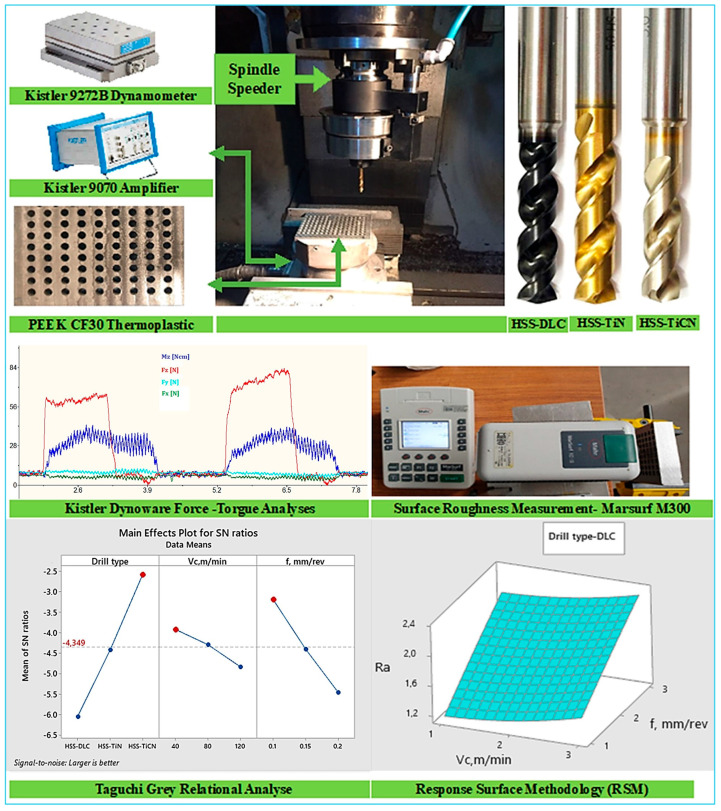
Experimental procedure and analysis of responses.

The variation in four machining outputs/responses (thrust force, surface roughness, drilling torque, and energy consumption) depending on drill coating quality and drilling parameters during drilling of PEEK-CF30 thermoplastic composite material was investigated in detail. PEEK-CF30 composite material is produced by adding short fiber-reinforced carbon (30%) to a polyether ether ketone matrix. This black material has a melting point of 340 °C and is supplied under the trade code Ketron^®^ CF30 PEEK (Mitsubishi Chemical Group, Tielt, Belgium). A workpiece of 75 × 100 × 10 mm material was used for drilling the polymer composite. Nachi brand DLC-, TiN-, and TiCN-coated HSS drills with a Ø5 mm, a 135° tip, and a 25° helix angle were used for drilling. The experiments were performed at a Johnford VMC-550 CNC (Roundtop Machinery Industries Co., Ltd., Taiwan, China) with a 1:4.8 gear ratio and a spindle speeder capable of reaching a maximum speed of 30,000 rpm. The thrust force (Fz) and drilling torque (Mz) generated during drilling were measured using a Kistler 9272 type dynamometer (Kistler Instruments Ltd., Winterthur, Switzerland), and a Kistler 5070A multi-channel amplifier (Kistler Instruments Ltd.) was used for data transmission. The measured data were analyzed in a computer environment using Kistler Dynoware software (Version 3.2.5.0). The roughness values of the hole surface were measured with a MarSurf M300 roughness device (Mahr GmbH, Göttingen, Germany), and the mean roughness value (Ra) was analyzed as the roughness indicator. In this operation, the cut-off and sampling distances were set to 0.8 mm and 4 mm. Measurements were taken four times, parallel to the hole axis, and the workpiece was rotated at an equal angle (90°) for each measurement. The arithmetic mean of the measurements was used for evaluation. The analysis and modeling processes applied for the responses and the experimental methods are presented in Figure 1.

During the experiments, cutting energy consumption (Ec) was calculated by considering only the cutting torque at the chip removal moment and the machining time (Tm, in s). In this process, machining time was taken as the time elapsed throughout the entry–exit distance of the drill bit into the workpiece (over 10 mm), and this drill movement was controlled by a CNC code. Average values obtained from the graphs in Dynoware software (Version 3.2.5.0) were used for cutting torque. In this stage, the cutting power (Pc, in watt) was first calculated according to the measured torque value. Consequently, the Ec (in J) values without auxiliary energy consumption were calculated using Equations (1)–(3).

(1) Pc=2π(Mz×N)/60

(2) Tm=L/(fz×N)

(3) Ec=Pc×Tm

### 2.2. Experimental Design

In drilling PEEK-CF30 composites, the parameters drill bit coating (Dc), cutting speed (Vc), and feed rate (fz) were selected. When selecting parameter levels, the drill supplier’s catalog was examined and parameter recommendations for drilling similar materials were identified. Preliminary experiments were conducted based on these parameters and levels, and the cutting speeds and feed rates used in the actual experiments were selected accordingly (Table 1). To contribute to sustainability and to facilitate adaptation to the TGRA methodology, drilling experiments were performed with the Taguchi L27 test series. In addition, to increase the validity of the predictive models developed for the responses (force, surface roughness, torque and energy consumption), experiments were repeated twice, and all assessments (effect analysis of parameter, optimization and modeling) were made by averaging the results.

### 2.3. Multi-Objective Optimization

Multi-objective optimization approaches play a fundamental role in determining the most appropriate process parameters to enhance efficiency while minimizing operational costs. Nevertheless, the simultaneous consideration of multiple responses introduces a major challenge, particularly in assigning appropriate weights to each output. Hybrid optimization strategies are frequently employed to address this issue. Among these, the Taguchi-based gray relational analysis (TGRA) method is widely implemented due to its effectiveness in handling multi-response problems. The optimization procedure implemented in this study follows the standard TGRA framework, as outlined below.

The initial stage of the TGRA method involves the normalization of experimental data. This step converts the measured response values into a dimensionless scale ranging from 0 to 1, allowing different responses with varying units to be evaluated on a common basis [39,40]. Depending on the desired performance criterion for each response, different normalization methods are applied using Equations (4)–(6).

The maximum is better,

(4) $$x_{ij}=\frac{y_{ij}-min(y_{ij})}{max(y_{ij})-min(y_{ij})}$$

The minimum is better,

(5) $$x_{ij}=\frac{maks(y_{ij})-y_{ij}}{max(y_{ij})-min(y_{ij})}$$

The nominal is better,

(6) $$x_{ij}=1-\frac{y_{ij}-y_{j}^{*}}{max(y_{ij})-min(y_{ij})}$$

In these formulations, $x_{ij}$ represents the normalized value corresponding to the original experimental data $y_{ij}$. The normalization process utilizes the min. and max. values within the dataset, denoted by $\min\left(y_{ij}\right)$ and $\max\left(y_{ij}\right)$, respectively. For cases where a specific target value is required, $y_{j}^{*}$ is introduced as the reference point. Following normalization, a reference sequence is defined, typically as $x_{0}=1$, and the gray relational coefficient (GRC) is calculated using Equation (7) to quantify the closeness between each normalized data point and the reference sequence.

(7) $$\gamma \left(x_{0j},x_{ij}\right)=\frac{\Delta_{min}+\zeta \Delta_{max}}{\Delta_{ij}+\zeta \Delta_{max}}$$

The gray relational coefficient, expressed as $\gamma \left(x_{0j},x_{ij}\right)$, evaluates the relational degree between the compared sequences. Here, $\Delta_{ij}=|x_{0j}-x_{ij}|$ signifies the absolute deviation between the reference and the normalized value, while $\Delta_{\min}$ and $\Delta_{\max}$ denote the minimum and maximum deviations within the dataset, respectively. The distinguishing coefficient ζ, which ranges between 0 and 1, is used to control the sensitivity of the analysis. Although variations in ζ may influence the numerical value of the GRC, its effect on the ranking of results is generally negligible; therefore, a value of 0.5 is commonly adopted [41,42]. Subsequently, the grey relational grade (GRG) is determined to represent the overall performance index of each experimental condition. The GRG is obtained by aggregating the individual GRC values associated with each response, as defined in Equation (8). A higher GRG value indicates a stronger correlation with the ideal performance condition.

(8) $$\Gamma \left(X_{0},X_{i}\right)=\sum_{j=1}^{n}w_{j}\gamma \left(x_{0j},x_{ij}\right)$$

(9) $$\sum_{j=1}^{n}w_{j}=1$$

In Equation (8), Γ denotes the GRG, which is calculated as a weighted sum of the GRC values, where $w_{j}$ represents the assigned weight of each response. When all responses are considered equally important, identical weights are assigned under the constraint that the total sum of weights equals unity.

Finally, the signal-to-noise (S/N) ratio is computed based on the GRG values to identify the optimal parameter levels. Since higher GRG values correspond to improved overall performance, the “larger-is-better” criterion is applied in the S/N ratio calculation using Equation (10). In this formulation, $y_{i}$ denotes the response of the i-th experiment, and n represents the total number of experiments.

(10) $$S/N=-10\log\left(\frac{1}{n}\sum_{i=1}^{n}\frac{1}{y_{i}^{2}}\right)$$

For proving the optimization results, confirmation experiments are done under the determined optimal parameters. The benchmarking between the experimental results and the predicted values provides an assessment of the reliability and accuracy of the TGRA-based optimization.

## 3. Findings and Discussion

### 3.1. Analysis of Responses

#### 3.1.1. Thrust Force

**Figure 2:**
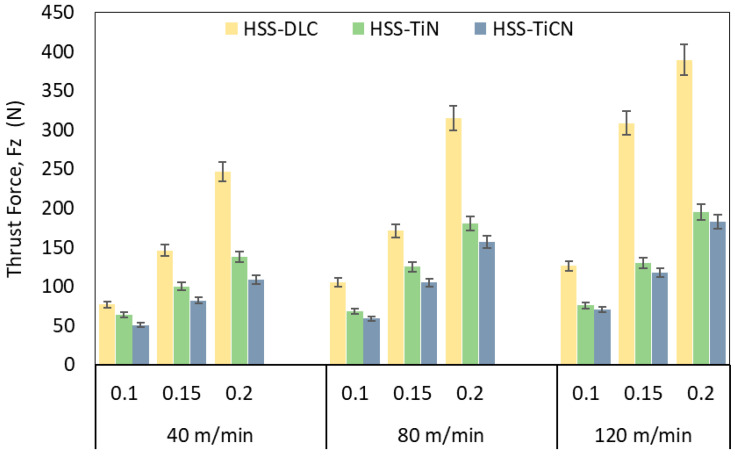
Thrust force versus cutting parameters.

**Figure 3:**
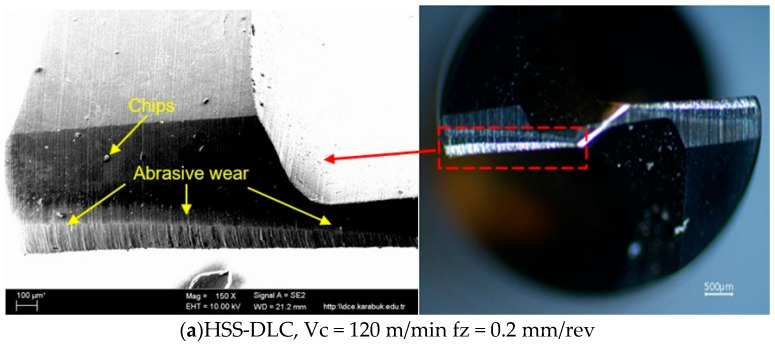

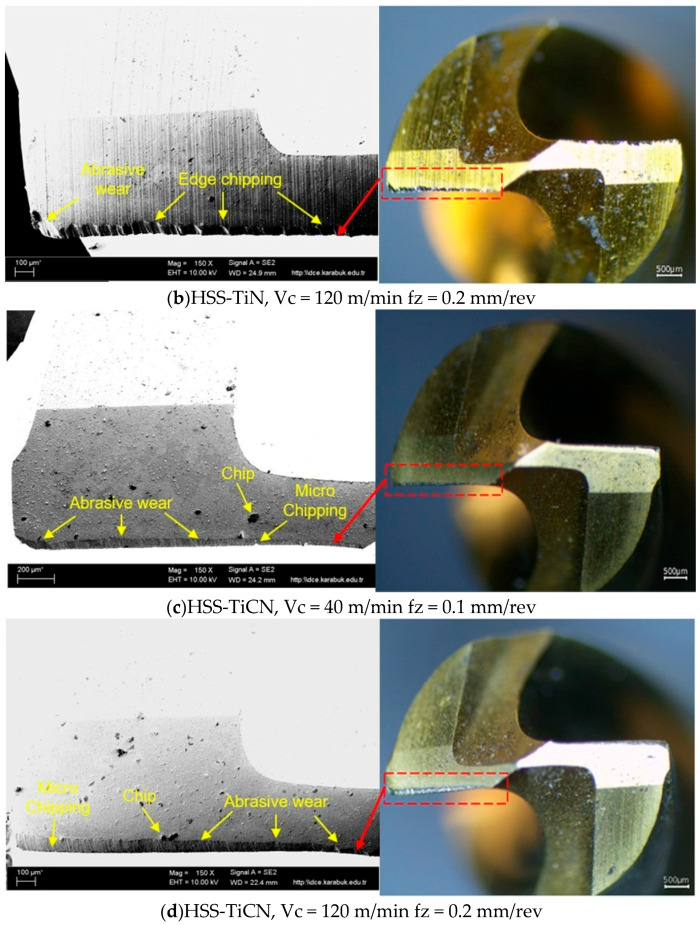
SEM and optical images for the coated HSS drills.

The change in thrust force with respect to feed rate and cutting speed in drilling operations performed by three different coated HSS drills is illustrated in Figure 2. As shown in Figure 2, the Fz values increased proportionally with the rise in cutting speed and feed rate. When the variation in Fz according to drill coating type was examined, the highest thrust force was obtained with the DLC-coated drill at a feed rate of 0.2 mm/rev and a Vc of 120 m/min, while the lowest thrust force was obtained with the TiCN-coated drill at a Vc of 40 m/min and a feed rate of 0.1 mm/rev. The high Fz value obtained with the DLC-coated drill is attributed to the abrasive wear on the drill, especially at high cutting speeds (Figure 3a). This result is attributed to the fact that, although DLC coatings are harder than other coating materials, they have lower impact resistance. Under the same cutting conditions, the TiC-coated drill shows more chipping (Figure 3b). The lower friction coefficient of the TiCN-coated drill compared to the others has resulted in less wear on this drill (Figure 3d). When the cutting speed increases, the increasing temperature triggers thermal softening in the matrix material and a higher tool–chip contact distance, significantly reducing the effective clearance angle [43]. This situation causes an increase in the friction coefficient, creating additional resistance at the tool–workpiece interface during drilling and resulting in a high thrust force. However, with improving cutting speed, the collision between the drill and the composite reinforcement element (short carbon fiber) leads to an abrasive wear mechanism and coating delamination [44]. Consequently, faster tool wear means a change in the drill’s geometry and a decrease in its cutting ability, resulting in an increase in the force Fz. The SEM images in Figure 3 support this phenomenon, which is even more pronounced in the DLC-coated drill. On the other hand, to remove the increased chip cross-section with increasing feed rate, the drill requires more cutting force, which leads to an increase in Fz values. Furthermore, as can be seen from Figure 3c,d, the increase in feed rate supports the increase in Fz by increasing drill cutting edge wear [45,46].

#### 3.1.2. Surface Roughness

**Figure 4:**
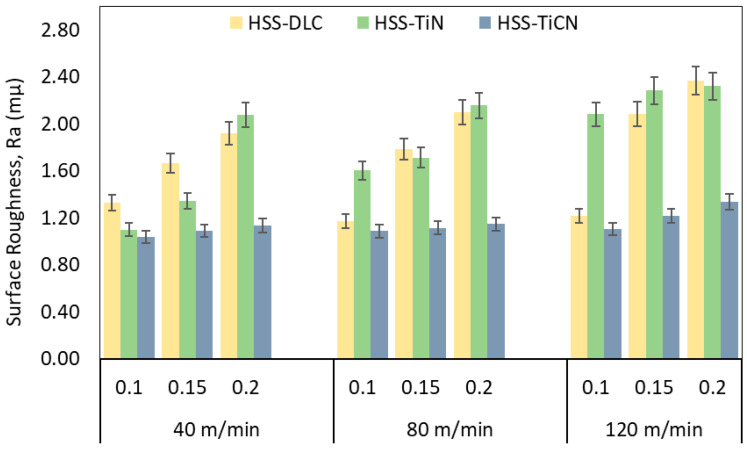
Surface roughness versus cutting parameters.

**Figure 5:**
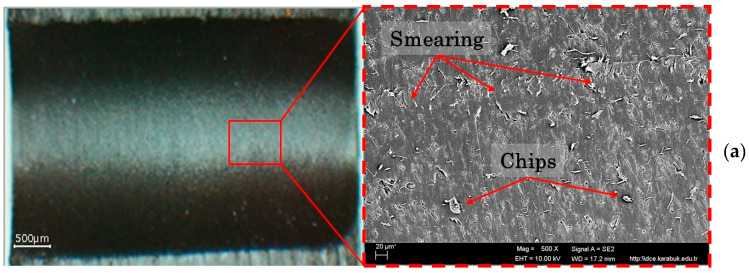

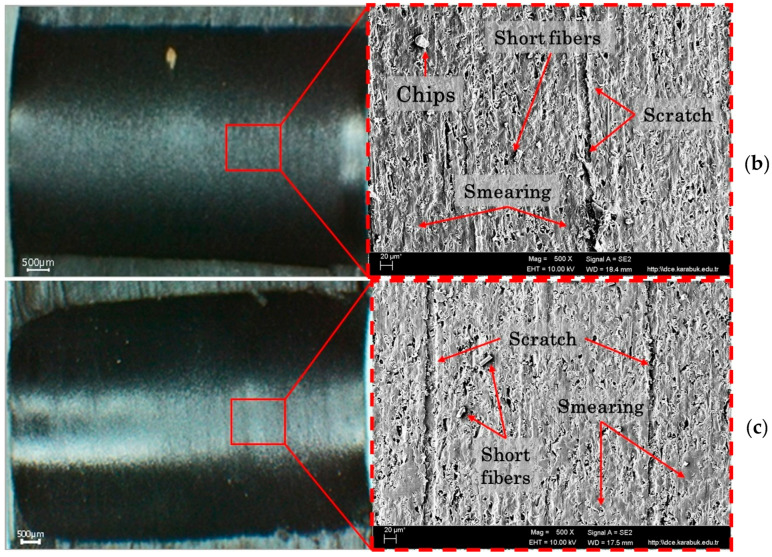
Hole surface images at Vc = 40 m/min fz = 0.1 mm/rev, (**a**) HSS-DLC, (**b**) HSS-TiN, (**c**) HSS-TiCN.

**Figure 6:**
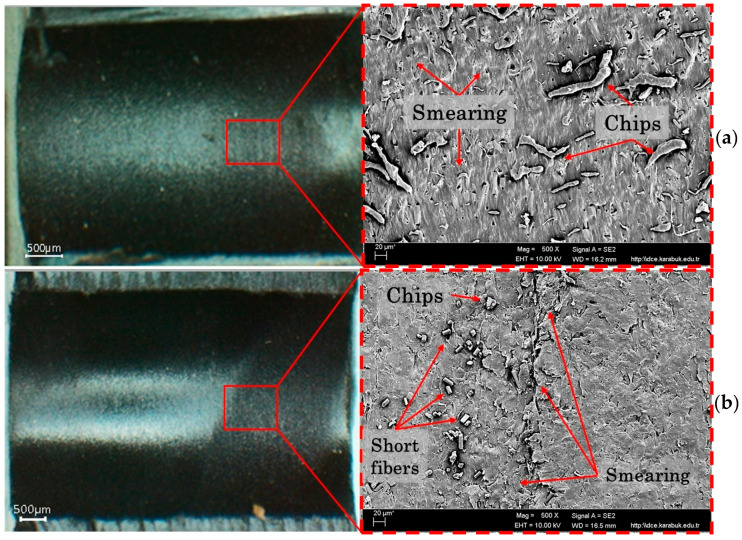

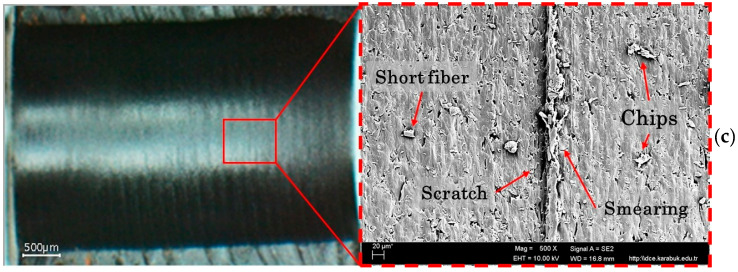
Hole surface images at Vc = 120 m/min fz = 0.2 mm/rev, (**a**) HSS-DLC, (**b**) HSS-TiN, (**c**) HSS-TiCN.

Figure 4 shows the change in surface roughness with respect to cutting speed and feed rate when drilling PEEK-CF30 composite using coated HSS drills. The lowest surface roughness (1.038 µm) was obtained with a TiCN-coated drill at a Vc of 40 m/min and a feed rate of 0.1 mm/rev, while the highest surface roughness (2.369 µm) was obtained with a DLC-coated drill at a Vc of 120 m/min and a feed rate of 0.2 mm/rev. Furthermore, lower surface roughness levels were achieved with the TiCN-coated drill bit at all cutting and feed rate levels. Increasing the cutting speed increases heat generation relative to friction resistance, while high feed rates reduce the contact time between the drill and workpiece, thus lowering the temperature in the cutting zone. While most of the heat created throughout the machining of metallic materials is removed with the chip, in polymeric materials, most of the heat is transferred to the tool and the machined part [47]. In this case, the increase in heat in the machined material causes more melting in the matrix, resulting in smearing on the hole surfaces. Due to the increasing temperature towards the end of the hole, the softened matrix is scratched by the sliding chips, with chip adhesion and short fiber pull-out resulting in poorer surface quality, as mentioned in the literature [46]. The SEM images in Figure 5 and Figure 6 supports these conclusions.

Furthermore, at high cutting speeds, the 30% CF in the material structure increases the damage caused by abrasive wear at the lips of the drill, making the cutting process more difficult [48]. In this case, as can be seen in Figure 6, more smearing, pull-out and chip adhesion occur on the drilled surface, causing the roughness to increase. On the other hand, although the decrease in temperature in the cutting zone due to the reduced tool-workpiece contact time at high feed rates reduces surface smearing, the excessive plastic deformation resulting from the increased chip cross-section causes more fibers to be removed from the matrix, leading to increased mechanical damage on the hole surfaces (Figure 6). Increasing the feed rate caused an increase in Ra in all coated HSS drills due to the above-mentioned surface formations, as seen in the SEM images. Therefore, as with the processing of other polymer-based composites, low cutting speed and feed rate are necessary for optimal surface quality when drilling PEEK-CF30 composite material.

#### 3.1.3. Drilling Torque

**Figure 7:**
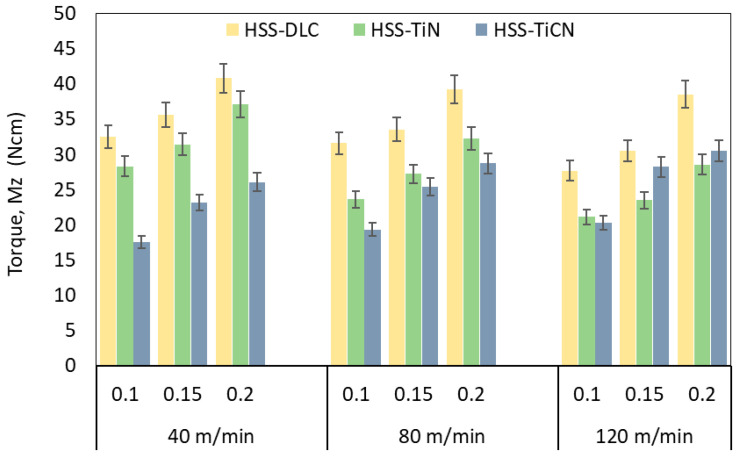
Torque versus cutting parameters.

Figure 7 shows the variation of torque amounts generated during drilling with three different coated drills, depending on the cutting speed and feed rate. When drilling PEEK-CF30 material, the highest torque was obtained with the DLC-coated drill (40.82 Ncm), while the minimum torque was obtained with the TiCN-coated drill (17.54 Ncm). It was observed that the torques generated during drilling with DLC-, TiN-, and TiCN-coated drills decrease as the cutting speed increases and increase as the feed rate increases. Increased friction between the tool and workpiece increases heat generation with increasing cutting speed or decreasing feed rate [49]. Additionally, torque is primarily affected by the cutting-edge radius and rake angle, and these tool geometries directly influence chip formation and thus the force components in drilling [50]. Therefore, as the cutting speed increases, the corresponding force component (in the Mz direction) decreases due to the increasing temperature, and consequently, the torque values also decrease. Since the thermal conductivity of PEEK-CF30 composite is lower than that of metals, elevated cutting temperatures trigger softening of the matrix material, resulting in a decrease in cutting torque. Conversely, although the friction constant of the DLC-coated drill is lower than that of other coated tools, the excessive wear produced by the abrasive mechanism in the DLC-coated drill leads to a change in the effective tool clearance angle [44], making plastic deformation more difficult and consequently increasing the torque. Similarly, the increasing chip volume with increasing feed rate made plastic deformation more difficult and subsequently increased the cutting force and torque, as reported in the literature [48,51].

#### 3.1.4. Energy Consumption

**Figure 8:**
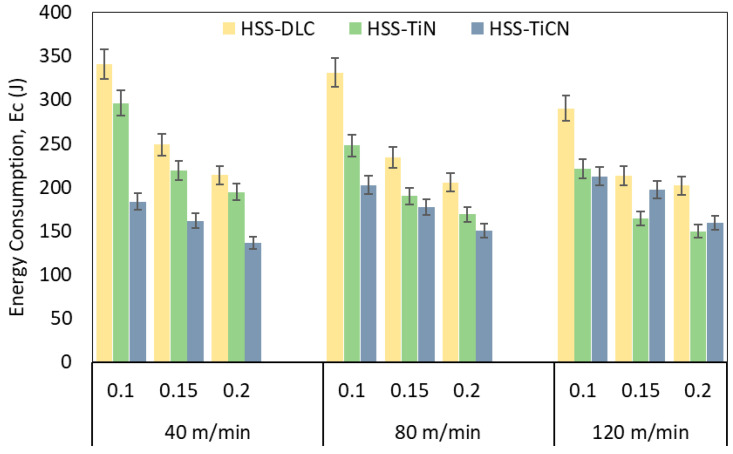
Energy consumption versus cutting parameters.

Sustainability in the machining sector is evaluated based on many responses such as energy consumption, tool life, surface integrity, cooling/lubrication, environmental factors, and the type of the raw material [52]. In this context, energy consumption is an essential output in drilling polymer-based composites due to the material’s durability and the required quality criteria in the machined material. The variation in energy consumption during drilling of PEEK-CF30 thermoplastic material with DLC-, TiN-, and TiCN-coated HSS drills as a function of cutting parameters is presented in Figure 8. It is observed that the total energy consumption (Ec) decreases with increasing cutting speed and feed rate in all three tool qualities. Theoretically, an increase in cutting speed means an increase in rotational speed and consequently an increase in the energy consumed by the machine tool [53,54]. However, the increase in temperature with increasing cutting speed leads to softening of the material and a decline in the energy consumption required for plastic deformation. As a result, the reduction in the specific cutting power requirement due to the cutting temperature reduces the thrust force, thus decreasing the Ec value. On the other hand, the contact time between the drill and the machined material decreases with increasing feed rate [55]. This phenomenon shortens the machining time, thus reducing the total energy consumption. As can be seen in Figure 8, energy consumption decreased proportionally with increasing feed rate while the cutting speed remained constant. The highest energy consumption occurred with the DLC-coated drill (340.59 J), while the lowest energy consumption occurred with the TiCN-coated drill (136.45 J). Similar to the theory mentioned above, the lowest Ec value was obtained at the maximum feed rate (0.2 mm/rev) and the minimum cutting speed (40 m/min) ). This shows that drill coating quality is a significant factor to consider regarding energy consumption and, indirectly, sustainability when drilling carbon/thermoplastic composites under the same cutting conditions.

### 3.2. Optimization by TGRA Method

The evaluation of the experimental results revealed that the optimal parameter settings differ for each individual response (Fz, Ra, Mz, and Ec), as discussed in the preceding sections. Since these responses are influenced simultaneously by the same process parameters, a combined optimization approach becomes necessary for achieving overall performance improvement. In addition, the differing units and scales of the responses make direct comparison impractical. For this reason, a hybrid optimization approach based on the TGRA method was employed.

Within this framework, the first step involved selecting an appropriate normalization scheme to convert all response values into a comparable dimensionless form. Considering that all responses were required to be minimized, the “smaller-is-better” normalization approach (Equation (4)) was applied to Fz, Ra, Mz, and Ec. After normalization, the gray relational coefficients (GRCs) were computed for each response using Equation (6). In the subsequent stage, the gray relational grade (GRG), which denotes the overall performance index, was determined using Equation (7).

Since all performance indicators were assumed to contribute equally to the overall evaluation, identical weighting factors were assigned to each response. Accordingly, the weight coefficient for each response was taken as $w_{j}=1/3$. The calculated GRC and GRG values for all experimental runs are summarized in Table 2.

To identify the optimal parameter combination, the S/N ratios of the GRG values were calculated based on the “larger-is-better” criterion (Table 3). In this examination, higher S/N ratios correspond to improved overall performance. The parameter level with the highest S/N ratio was therefore considered optimal, while the delta values were used to rank the relative influence of each factor.

**Figure 9:**
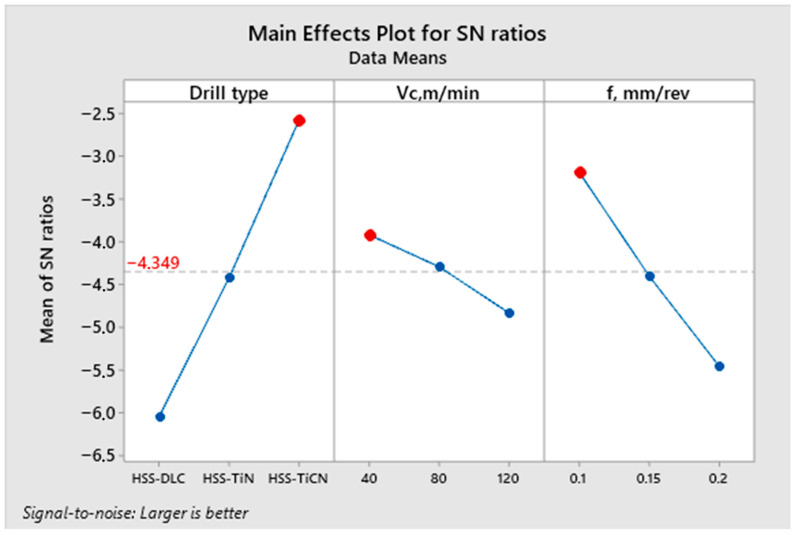
S/N ratio plot for GRG.

Based on this evaluation, the best parameter sequence was determined as Dc3–Vc1–fz1, corresponding to the TiCN-coated drill, a Vc of 40 m/min, and a fz of 0.1 mm/rev. The validity of these optimal conditions was further supported by the S/N ratio plots presented in Figure 9, where the peak values indicate the best parameter levels. According to the principles of the Taguchi method, when the identified optimal parameter combination already exists within the experimental plan, additional confirmation experiments are not mandatory [41]. However, to ensure the reliability of the results, a confirmation experiment was conducted using the determined optimum combination, and the results were compared (Table 4). Table 4 shows that the difference between the predicted and experimental results is less than 5%. This result indicates that the optimum parameters found using the TGRA methodology can be reliably used in the sustainable drilling of PEEK-CF30.

### 3.3. Modeling by RSM

Response surface methodology (RSM) is extensively employed as an effective statistical and mathematical framework for modeling and optimization in engineering applications. It is particularly advantageous in machining studies, where it enables systematic investigation of the influence of process parameters on performance outputs while supporting more efficient and environmentally conscious process design. By establishing functional relationships between input variables and responses, RSM facilitates both optimization and interpretability of complex process behavior [56,57].

The methodology generally proceeds through a sequence of structured steps. Initially, a set of experiments is designed and conducted to obtain response data corresponding to selected combinations of process parameters. Subsequently, an empirical model is formulated—most commonly in the form of a second-order polynomial—to describe the relationship between the independent variables and the measured responses. This regression-based model allows for reliable estimation of response values within the experimental domain, including intermediate conditions that were not directly tested. In the final stage, the developed model is analyzed using statistical and graphical tools such as analysis of variance (ANOVA), response surface plots, and Pareto charts to evaluate the significance of parameters and their interactions. In the present study, quadratic regression models were employed to represent the connections between the input parameters and the responses. The general second-order RSM model can be stated as follows [42]:

$$Y_{i}=\beta_{0}+\sum_{i=1}^{k}\beta_{i}x_{i}+\sum_{ij}^{k}\beta_{ij}x_{i}x_{j}\sum_{i=1}^{k}\beta_{ii}x_{i}^{2}+\varepsilon_{ij}$$

Here i, j, k = 1, 2 … n. In this formulation, $\beta_{0}$ denotes the intercept term, while $\beta_{i}$, $\beta_{ij}$, and $\beta_{ii}$ denote the linear, interaction, and quadratic regression coefficients, respectively. The parameters $x_{i}$ and $x_{j}$ correspond to the independent process parameters, and ε accounts for the experimental error. The dependent variable $Y_{i}$ represents the responses considered in this study, namely thrust force, surface roughness, torque, and energy consumption.

The mathematical models acquired for the responses (*Fz*, *Ra*, *Mz*, *Ec*) are given as equations in Table 5. The coefficient of determination (*R*^2^) is considered to control the consistency of the predictive models [58]. Accordingly, the values of *R*^2^ indicator for *Fz*, *Ra*, *Mz*, and *Ec* of the models developed for HSS drills with different coating qualities (*DLC*, *TiN*, and *TiCN*) are 97.75%, 93.59%, 97.75% and 97.25%, respectively.

**Figure 10:**
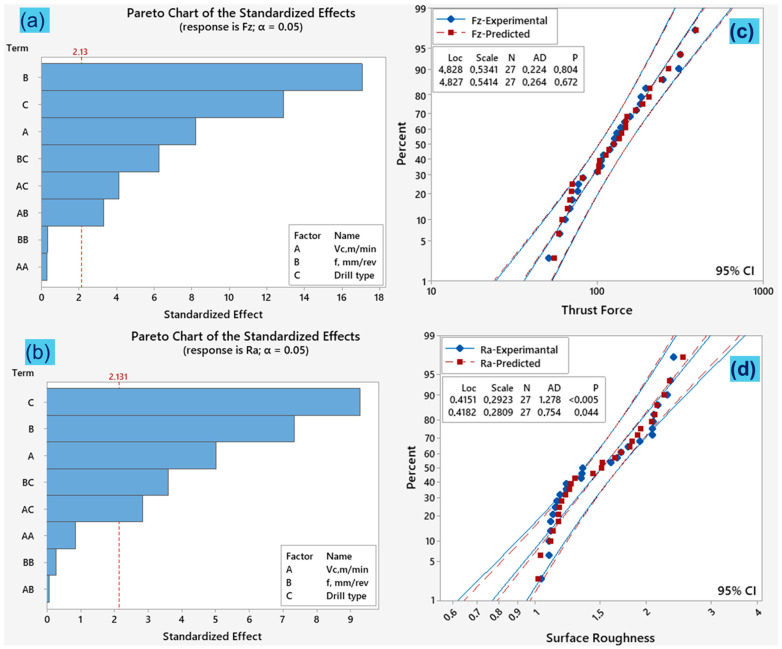
For Fz and Ra models: (**a**,**b**) Pareto charts. (**c**,**d**) Probability plots.

**Figure 11:**
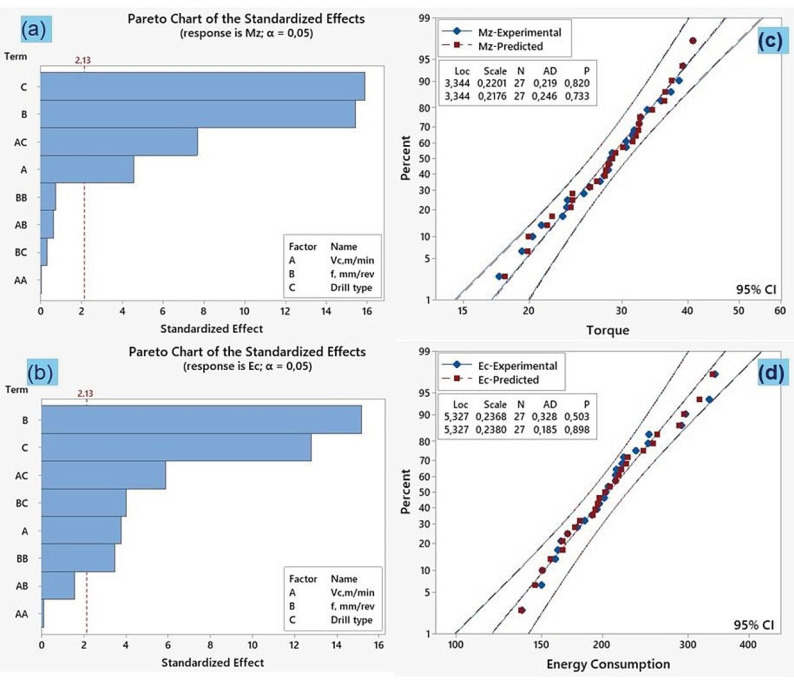
For Mz and Ec models: (**a**,**b**) Pareto charts; (**c**,**d**) Probability plots.

The Pareto plot of standardized effects provides the statistical significance of specific variables (terms) in the predictive model [59]. Thus, Figure 10a,b and Figure 11a,b show the Pareto charts used to analyze the degree of the impacts of the parameters on *Fz*, *Ra*, *Mz* and *Ec* at a 95% significance level. The dotted line with a standardized effect of 2.13 in Figure 10 and Figure 11 indicates the cut-off point for identifying terms that are statistically significant at a 95% confidence level. The standardized effects indicate that feed rate (B) has the most significant parameter for thrust force and energy consumption while drill type (C) has the most dominant parameter for torque and surface roughness at the linear level. At the interaction effect of the parameters, feed rate*drill type (BC) is the most important for thrust force and surface roughness while cutting speed*drill type (AC) is essential for torque and energy consumption.

The experimental and predicted data for the responses (*Fz*, *Ra*, *Mz*, *Ec*) are also compared via normal probability graphics. Figure 10c,d and Figure 11c,d show the goodness of fit with the representation of the predicted data against the experimental data for each RSM model. This is evident from the fact that the data distributions predicted by the mathematical models and experiments are close to a straight line, as reported in the literature [60]. The graphs show that the errors in the resulting formulations have a normal distribution. These outcomes show the reliability and applicability of the predictive models developed using response surface methodology in drilling of PEEK-CF30 composite.

## 4. Conclusions and Suggestions

The thrust force, surface roughness, torque, and energy consumption associated with the drilling behavior of PEEK-CF30 composites were investigated by considering the combined influence of the drill coating type and cutting parameters. The experimental findings, optimization results, and predictive modeling results are summarized below:

1. In all coated drills, higher thrust force was measured with corresponding increases in cutting speed and feed rate. The maximum Fz was obtained using a DLC-coated drill at a Vc of 120 m/min and a fz of 0.2 mm/rev. This result was attributed to increased chip load at high feed rates and abrasive wear caused by short carbon fibers during tool–workpiece interaction at high cutting speeds. Surface quality was degraded as a result of smearing and adhesion of chips or short fibers to the surface during drilling due to tool wear. TiCN-coated drills provided lower roughness values compared to DLC- and TiN-coated tools within the tested parameter range. The lowest Ra was measured as 1038 µm with this drill at a Vc of 40 m/min and a fz of 0.1 mm/rev.
2. Drilling torque decreased with increasing cutting speed, while it increased with increasing feed rate in all drills. At high parameter levels, wear in the drills altered the tool geometry, disrupting plastic deformation stability and resulting in higher torque values. The lowest Mz was measured with the TiCN-coated drill at a Vc of 40 m/min and a fz of 0.1 mm/rev.
3. Energy consumption decreased due to the reduced machining time with increasing feed rate and the lower cutting force demand for plastic deformation resulting from matrix softening provided by increasing cutting speed across all drill qualities. The lowest energy requirement was observed with TiCN-coated drills, while the highest energy requirement was observed with DLC-coated drills.
4. Taguchi GRA optimization results showed that the ideal drilling combination is a TiCN-coated HSS drill with a cutting speed of 40 m/min and a feed rate of 0.1 mm/rev. Furthermore, ANOVA results indicated that the drill coating type was the most important parameter, followed by feed rate and cutting speed.
5. The R^2^ values of 97.75% for thrust force, 93.59% for surface roughness, 97.75% for torque, and 97.25% for energy consumption obtained from the prediction models developed using RSM indicate that the equations can be used to predict the drilling performance of PEEK-CF30 material. According to the Pareto analysis, feed rate was the main determinant of thrust force and energy consumption, while drill type had the greatest effect on torque and surface roughness.
6. Overall, results indicate that drill coating is an effective factor in terms of sustainability when drilling polymer-based composites. Future studies could focus on the effects of different cutting environments on the machining outputs examined in the presented study, as well as on parameter optimization and the development of prediction models for drill life and hole dimensional accuracy.

## Figures and Tables

**Table 1:** Parameters for experimental design.

Parameter	Dc	Vc (m/min)	fz (mm/rev)
Level 1	HSS-DLC	40	0.1
Level 2	HSS-TiN	80	0.15
Level 3	HSS-TiCN	120	0.2

**Table 2:** Taguchi GRA findings.

Exp. No	Exp. Results				Normalization				GRC				GRG	Order
	Fz	Ra	Mz	Ec	Fz	Ra	Mz	Ec	Fz	Ra	Mz	Ec		
1.	76.70	1.332	32.51	340.59	0.924	0.779	0.357	0.000	0.8675	0.6936	0.4374	0.3333	0.5830	17
2.	146.05	1.667	35.62	248.78	0.719	0.527	0.223	0.450	0.6400	0.5141	0.3917	0.4761	0.5055	21
3.	246.9	1.921	40.82	213.82	0.421	0.337	0.000	0.621	0.4634	0.4298	0.3333	0.5688	0.4488	25
4.	105.45	1.171	31.60	331.05	0.839	0.900	0.396	0.047	0.7561	0.8334	0.4529	0.3441	0.5966	16
5.	171.25	1.788	33.52	234.11	0.644	0.437	0.314	0.522	0.5844	0.4702	0.4214	0.5110	0.4967	22
6.	315.3	2.101	39.23	205.49	0.219	0.201	0.068	0.662	0.3903	0.3850	0.3492	0.5965	0.4303	26
7.	126.35	1.218	27.70	290.19	0.777	0.865	0.564	0.247	0.6916	0.7871	0.5339	0.3990	0.6029	14
8.	308.65	2.087	30.51	213.08	0.239	0.212	0.443	0.625	0.3964	0.3882	0.4730	0.5712	0.4572	24
9.	389.4	2.369	38.52	201.77	0.000	0.000	0.099	0.680	0.3333	0.3333	0.3568	0.6098	0.4083	27
10.	63.85	1.101	28.30	296.48	0.962	0.953	0.538	0.216	0.9287	0.9135	0.5196	0.3894	0.6878	8
11.	99.8	1.348	31.41	219.37	0.855	0.767	0.404	0.594	0.7757	0.6822	0.4563	0.5518	0.6165	13
12.	138.05	2.080	37.15	194.59	0.742	0.217	0.158	0.715	0.6600	0.3898	0.3725	0.6371	0.5148	20
13.	68.1	1.604	23.66	247.86	0.949	0.575	0.737	0.454	0.9075	0.5404	0.6554	0.4781	0.6454	10
14.	125.1	1.713	27.22	190.11	0.781	0.493	0.584	0.737	0.6951	0.4965	0.5460	0.6554	0.5982	15
15.	180.8	2.157	32.30	169.19	0.616	0.159	0.366	0.840	0.5657	0.3729	0.4409	0.7571	0.5342	19
16.	75.6	2.082	21.13	221.36	0.927	0.216	0.846	0.584	0.8724	0.3893	0.7643	0.5459	0.6430	11
17.	130.2	2.285	23.51	164.19	0.766	0.063	0.744	0.864	0.6808	0.3480	0.6610	0.7863	0.6190	12
18.	194.95	2.323	28.56	149.60	0.574	0.035	0.527	0.936	0.5402	0.3412	0.5137	0.8859	0.5702	18
19.	50.85	1.038	17.54	183.76	1.000	1.000	1.000	0.768	1.0000	1.0000	1.0000	0.6833	0.9208	1
20.	82.05	1.089	23.17	161.82	0.908	0.961	0.758	0.876	0.8444	0.9283	0.6740	0.8009	0.8119	3
21.	108.4	1.135	26.05	136.45	0.830	0.927	0.634	1.000	0.7463	0.8728	0.5777	1.0000	0.7992	4
22.	58.9	1.089	19.35	202.71	0.976	0.962	0.922	0.675	0.9546	0.9288	0.8654	0.6064	0.8388	2
23.	104.95	1.116	25.41	177.46	0.840	0.941	0.662	0.799	0.7578	0.8951	0.5966	0.7134	0.7407	6
24.	156.45	1.148	28.72	150.44	0.688	0.917	0.520	0.931	0.6158	0.8582	0.5101	0.8795	0.7159	7
25.	70.4	1.105	20.28	212.45	0.942	0.950	0.882	0.628	0.8965	0.9085	0.8095	0.5732	0.7969	5
26.	117.85	1.217	28.24	197.23	0.802	0.866	0.540	0.702	0.7164	0.7880	0.5210	0.6268	0.6631	9
27.	182.5	1.339	30.51	159.81	159.81	0.774	0.443	0.886	0.0031	0.6886	0.4730	0.8138	0.4946	23

**Table 3:** Response table for GRG.

Parameter	1	2	3	Delta	Rank
Dc	−6.048	−4.424	−2.574	3.474	1
Vc	−3.918	−4.293	−4.834	0.916	3
fz	−3.186	−4.402	−5.458	2.272	2
Mean of GRG = −4.349					

**Table 4:** Comparison for predicted and confirmation experiment.

Response	Predicted	Experiment	Error (%)
Fz	51.75	50.45	2.51
Ra	1.056	1.032	2.27
Mz	17.85	17.26	3.31
Ec	185.8	182.56	1.74

**Table 5:** Predictive models developed with RSM for the responses.

Responses	Predictive Models
Thrust force *R*^2^ = 97.75%	${Fz}_{DLC}=-45.3+19Vc+66.8f+{2Vc}^{2}+2.06f^{2}+16.05Vc\times f$ ${Fz}_{TiN}=62.2-23.6Vc+10.7f+{2Vc}^{2}+2.06f^{2}+16.05Vc\times f$ ${Fz}_{TiCN}=49.1-18.5Vc+4.2f+{2Vc}^{2}+2.06f^{2}+16.05Vc\times f$
Surface roughness *R*^2^ = 93.59%	${Ra}_{DLC}=0.706-0.082Vc+0.52f+{0.0535Vc}^{2}-0.0171f^{2}-0.0033Vc\times f$ ${Ra}_{TiN}=0.652+0.153Vc+0.37f+{0.0535Vc}^{2}-0.0171f^{2}-0.0033Vc\times f$ ${Ra}_{TiCN}=0.987-0.141Vc+0.14f+{0.0535Vc}^{2}-0.0171f^{2}-0.0033Vc\times f$
Torque *R*^2^ = 97.75%	${Mz}_{DLC}=31.69-2.4Vc+2.5f-{0.027Vc}^{2}+0.373f^{2}+0.234Vc\times f$ ${Mz}_{TiN}=29.81-4.3Vc+2.19f-{0.027Vc}^{2}+0.373f^{2}+0.234Vc\times f$ ${Mz}_{TiCN}=12.99+1.69Vc+2.73f-{0.027Vc}^{2}+0.373f^{2}+0.234Vc\times f$
Energy consumption *R*^2^ = 97.25%	${Ec}_{DLC}=473.1-24.3Vc-132.9f-{0.64Vc}^{2}+16.41f^{2}+5.26Vc\times f$ ${Ec}_{TiN}=422-37.2c-118.2f-{0.64Vc}^{2}+16.41f^{2}+5.26Vc\times f$ ${Ec}_{TiCN}=271+6.6Vc-101.5f-{0.64Vc}^{2}+16.41f^{2}+5.26Vc\times f$

## Data Availability

The raw data supporting the conclusions of this article will be made available by the first author on request.
